# Investigation and Rapid Determination of Storage-Induced Multidimensional Quality Deterioration of Wenyujin Rhizoma Concisum

**DOI:** 10.3390/foods15020274

**Published:** 2026-01-12

**Authors:** Haihui Liu, Tianze Xie, Jiaru Wang, Chuning Wei, Chenxiaoning Meng, Zhimin Wang, Jingjing Zhu

**Affiliations:** 1 National Engineering Laboratory for Quality Control Technology of Chinese Herbal Medicine, Institute of Chinese Materia Medica, China Academy of Chinese Medical Sciences, No. 16, Nanxiaojie, Dongzhimennei, Beijing 100700, China; 2 Institute of Chinese Materia Medica, Shanghai University of Traditional Chinese Medicine, Shanghai 201203, China; wangjiaru02@163.com; 3 XUTELI School, Beijing Institute of Technology, Beijing 100081, China

**Keywords:** Wenyujin Rhizoma Concisum, storage, functional food, bioactivity, sensory, hyperspectral imaging, machine learning

## Abstract

Prolonged storage degrades the quality of Wenyujin Rhizoma Concisum (PJH), a functional food ingredient rich in volatile bioactive terpenes, calling for an investigation and a rapid non-destructive identification method. This study adopts a holistic “bioactivity–composition–sensory” approach to evaluate PJH over time, combining cell assays, high-performance liquid chromatography, electronic nose, and hyperspectral imaging. Results show that extended storage leads to marked declines in anti-inflammatory and antioxidant activities, along with reductions in key volatile terpenes, weakened aroma, and color fading. A machine learning model was subsequently constructed based on hyperspectral data for storage year discrimination with 100% accuracy. These findings systematically reveal the multidimensional quality deterioration of PJH and establish a scientific basis for determining its shelf life. This holistic perspective and hyperspectral machine learning approach in this study offer a paradigm applicable to quality monitoring and stability research of other volatile-rich functional ingredients.

## 1. Introduction

Wenyujin Rhizoma Concisum, also known as Pianjianghuang (PJH), is processed from the rhizome of *Curcuma wenyujin* Y. H. Chen and C. Ling, and serves as a significant raw material for functional foods. Cultivation of *C. wenyujin* is concentrated in Zhejiang, where it has evolved into a scaled planting and processing industry chain. PJH production is a vital pillar of the local agricultural economy, supplying high-quality natural raw materials for numerous health products. Ensuring its quality stability throughout the production-to-consumption chain is of significant socio-economic value for promoting the healthy food industry and safeguarding the farmers’ income. Its diverse health-promoting properties, including anti-inflammatory, antioxidant, and antimicrobial activities, are primarily attributed to two major classes of bioactive compounds: curcuminoids and terpenes [1,2]. Among these, terpene components such as curdiol and germacrone not only constitute the core of its characteristic flavor profile, but also function as key contributors to its bioactivity [3]. Moreover, these terpenes form the essence of PJH’s distinctive “pungent, bitter, and warm” flavor. The unique aroma serves as a crucial sensory marker for consumers to identify quality, often evoking a sense of trust in natural, health-promoting ingredients. This distinctive sensory profile makes PJH an important cultural bridge connecting traditional dietary therapy concepts with modern health demands. However, these components are mostly volatile substances characterized by low boiling points and chemical instability, rendering them susceptible to evaporation, oxidation, and transformation during storage, which directly compromises the quality, stability and functional integrity of PJH [4,5].

Storage/shelf-life management is a crucial link in the food chain from production to consumption, directly influencing the flavor, functionality, edible safety, and commercial value of food. For PJH, there is currently no clearly defined, unified statutory shelf-life. In practice, quality assessment often relies on empirical judgment, and the risk of quality fluctuation increases due to environmental factors throughout production, transportation, and sales. Therefore, a systematic research into its quality change during storage is of great practical significance. Volatile flavor compounds, as important bioactive constituents in foods, have stability issues that directly influence the overall quality and shelf life of the product. These processes lead to the attenuation of component contents, flavor deterioration, and ultimately the reduction in the food’s edible value and functionality [6,7], as is seen in the examples of fried pepper and *Zanthoxylum bungeanum* [8,9]. Currently, research on the storage period of raw materials for functional food ingredients mostly focuses on changes in single indicator components, lacking a systematic and comprehensive evaluation of multi-dimensional quality indicators such as flavor, color, and functional activity [10]. Therefore, establishing a method to extensively and rapidly assess the comprehensive quality changes in raw materials during storage is crucial for ensuring the quality of food raw materials, particularly the stability and persistence of volatile flavor compounds.

Conventional methods for identifying storage periods mainly rely on artificial sensory evaluation and chemical analysis. Although “odor discrimination” by experienced practitioners reflects the wisdom of holistic evaluation, it has limitations such as strong subjectivity and poor reproducibility [11]. While chemical analysis methods such as high-performance liquid chromatography (HPLC) can accurately determine specific components, they are destructive, costly, and time-consuming, making it difficult to meet the needs of the modern food industry for rapid, non-destructive, and high-throughput screening of large quantities of raw materials [12]. Thus, the development of a rapid, accurate, and non-destructive detection technology at the raw material level has become an urgent demand in the field of food quality control.

In recent years, emerging non-destructive detection technologies have provided new insights into the quality evaluation of food raw materials. The Electronic Nose (E-nose) technology, which simulates the human olfactory system through a sensor array, can objectively and digitally capture the overall odor fingerprint of food, enabling stable and rapid digital characterization, making it particularly suitable for the analysis of volatile flavor components [13,14]. It possesses the advantages of rapidity, sensitivity, and non-destructiveness, and can overcome the limitation of vague traditional sensory descriptions. Numerous studies have confirmed its successful application in quality assessment, freshness evaluation, and aroma classification of foods such as tea, fish, and pomegranate [15,16,17]. For example, Xu et al. successfully evaluated tea quality by integrating E-nose, E-tongue, and E-eye with chemometrics; Buratti et al. developed E-nose-based control charts for fish freshness monitoring; and Zhang et al. identified key aroma substances in pomegranates from different geographical origins using volatile profiling combined with multivariate statistical analysis. These studies provide solid scientific evidence for applying the E-nose in monitoring the flavor deterioration of PJH during storage.

Meanwhile, Hyperspectral Imaging (HSI) integrates spectroscopy and imaging technologies to simultaneously acquire spatial and spectral information of samples, allowing comprehensive characterization of internal and external qualities with the merits of rapidity, non-destructiveness, and high throughput [18,19,20]. The introduction of Machine Learning (ML) algorithms, such as Support Vector Machine (SVM), Random Forest (RF), and Partial Least Squares–Discriminant Analysis (PLS-DA), enables efficient processing of high-dimensional and non-linear data generated by HSI, exhibiting significant potential in food quality monitoring and grading [21,22,23]. For instance, the combination of HSI and ML has achieved favorable results in green tea storage period identification, corn freshness evaluation, and *Coix* seed storage period discrimination [20,24,25]. Integrating non-destructive technologies like the E-nose and HSI into raw material shelf-life management represents a crucial step towards intelligent and standardized food industry practices, facilitating high-throughput, rapid, and online screening of raw material quality.

While the aforementioned technologies have enabled the detection of specific quality indicators in food, current research remains focused on single-technology analysis of limited indicators, and a systematic evaluation strategy for the multi-dimensional quality (e.g., flavor, functional activity, etc.) of food during storage has not been demonstrated.

Herein, this study hypothesizes that PJH undergoes systematic changes in the comprehensive quality (bioactivity, chemical composition, and sensory attributes) during storage, which can be rapidly and accurately characterized through integration of modern non-destructive techniques (E-nose and HSI) with ML. This study is grounded in the holistic perspective of “bioactivity–chemical composition–sensory attributes”, aiming to systematically investigate the impact of storage periods on the comprehensive quality of PJH. By innovatively integrating modern sensory analytical technologies with artificial intelligence, this study establishes a rapid and non-destructive identification model for the storage period of PJH. The specific research contents include (1) analyzing the changes in functional activity of PJH with different storage periods through in vitro antioxidant and anti-inflammatory activity evaluation systems, and correlating the retention law of functionality; (2) quantitatively determining the content of main functional components (terpenes) using HPLC to reveal the influence of storage periods on the material basis of functionality; (3) digitally characterizing and visually analyzing the flavor characteristics of samples with different storage periods via E-Nose technology, and correlating the changes in sensory quality; (4) collecting hyperspectral images of samples to construct a grade spectral dataset, and characterizing the dynamic changes in color and physicochemical properties; and (5) constructing and optimizing a high-precision identification model for the storage period of PJH based on various ML algorithms. This study intends to provide a scientific basis for the quality evaluation and shelf life determination of functional food raw materials such as PJH, and offer new methods and ideas for the development of intelligent, non-destructive, and rapid quality monitoring technologies in food research. This study offers a proof of concept on limited samples from a single core production area, laying the foundation for further explorations, including the incorporation of multi-region samples and large-scale industrial validation. Subsequent work can further improve the model stability to satisfy the practical quality control demands of production.

## 2. Materials and Methods

### 2.1. Materials and Reagents

PJH samples were collected from Wenzhou City, Zhejiang Province, China, comprising 200 samples from each of three different storage years (2025, 2024, and 2023), totaling 600 samples. Samples were stored under standard indoor conditions with a temperature of 25 °C, in a dry environment, and protected from light (avoiding direct sunlight, in compliance with industrial light-proof packaging specifications).

Ethanol and methanol (analytical grade) were purchased from Beijing Chemical Works; chromatographic grade methanol and acetonitrile were obtained from Fisher Scientific (Waltham, MA, USA); Wahaha purified water was provided by Hangzhou Wahaha Group Co., Ltd. (Hangzhou, China); zedoarondione reference standard was laboratory-purified (purity ≥ 98% by HPLC).

DMEM high-glucose medium, fetal bovine serum (FBS), and PBS buffer were all purchased from Gibco (Grand Island, NY, USA); CCK-8 cell proliferation assay kit was obtained from Dalian Meilun Biotechnology Co., Ltd. (Dalian, China); lipopolysaccharide (LPS, L2630) was purchased from Sigma (St. Louis, MO, USA); chemical reagents such as N-(1-naphthyl)ethylenediamine dihydrochloride, phosphoric acid, and sulfanilamide were obtained from Sinopharm Chemical Reagent Co., Ltd. (Shanghai, China); quantitative enzyme-linked immunosorbent assay (ELISA) kits for interleukin-6 (IL-6, VAL604G), tumor necrosis factor-*α* (TNF-*α*, VAL609), and interleukin-1*β* (IL-1*β*, VAL601) were purchased from Novus (Centennial, CO, USA). The PJH extracts (abbreviated as Ext.P) were self-prepared in the laboratory. The RAW 264.7 cell line was provided by the Cell Bank of the Chinese Academy of Sciences (Beijing, China).

### 2.2. In Vitro Functional Activity Evaluation Based on LPS-Induced RAW 264.7 Cell Model

#### 2.2.1. Preparation of PJH Extracts

PJH powder (100 g per storage year) was extracted twice by reflux with 95% ethanol (1:10, solid-to-liquid ratio). The extracts were concentrated under reduced pressure to obtain PJH extracts from different years, designated as Ext.P_25_ (2025), Ext.P_24_ (2024), and Ext.P_23_ (2023).

#### 2.2.2. Cell Culture and Viability Assay

RAW 264.7 mouse macrophages were cultured in DMEM supplemented with 10% FBS at 37 °C with 5% CO_2_. Cells in the logarithmic growth phase were used for experiments.

Cytocompatibility was assessed using the CCK-8 assay. Cells were seeded in 96-well plates (2 × 10^5^ cells/mL), treated with various concentrations of extracts (6.25–400 μg/mL) for 24 h, and then incubated with CCK-8 reagent for 1 h. Absorbance was measured at 450 nm, and the safe concentration threshold was defined as cell viability ≥ 80%.

#### 2.2.3. Assessment of Anti-Inflammatory Activity

To evaluate anti-inflammatory effects, cells were divided into control, LPS model, and sample treatment groups (12.5, 25, 50 μg/mL). The inflammation model was established by stimulating cells with LPS (1 μg/mL) for 24 h, concurrently with extract treatment. The culture supernatant was subsequently collected.

Nitric oxide (NO) production was quantified using the Griess method. The levels of TNF-*α*, IL-6, and IL-1*β* were determined using commercial ELISA kits according to the manufacturers’ protocols.

### 2.3. Determination of Terpene Functional Components in PJH with Different Storage Years

Zedoarondione reference standard was accurately weighed, dissolved in methanol, and serially diluted to prepare standard stock solutions with concentrations of 0.1, 0.2, 0.4, 0.8, and 1.6 mg/mL. Exactly 0.5 g of PJH sample powder was accurately weighed, mixed with 10 mL of methanol, and subjected to ultrasonic-assisted extraction for 30 min. The extract was filtered through a 0.22 μm microporous membrane, and the resulting filtrate was used as the HPLC analysis sample.

Analysis was performed on a Waters e2695/e2998 HPLC system (Waters Corporation, Milford, MA, USA). Chromatographic separation was performed on a Diamonsil C18 column (250 mm × 4.6 mm, 5 μm) at 35 °C. The detection wavelength was set at 244 nm, the flow rate was 1.0 mL/min, and the injection volume was 10 μL. The mobile phase consisted of a gradient system of acetonitrile (A) and water (B): 0–10 min (20% A), 10–35 min (20–46% A), 35–50 min (46–48% A), 50–55 min (48–54% A), 55–70 min (54–90% A), 70–75 min (90–95% A), and 75–85 min (95% A).

Based on the quantitative analysis of multi-components by the single marker (QAMS) method, nine functional terpene components in PJH were simultaneously determined: zedoarondiol, isozedoarondiol, aerugidiol, (4*S*,5*S*)-germacrone-4,5-epoxide, curcumenone, neocurdione, curdione, germacrone, and furanodinene [26]. This study systematically evaluated the changes in the content of these functional components as a food raw material.

### 2.4. Flavor Characteristic Analysis of Samples with Different Storage Periods

A JN03-type E-nose system (Juxin Zhuifeng, Beijing, China) was employed for the digital characterization of volatile flavor components in the samples. This system integrates 17 metal oxide sensors, with the specificity of each sensor as follows: 1 (olefins), 2 (aldehydes and alcohols, high threshold), 3 (organic sulfides), 4 (aldehydes and alcohols, medium threshold), 5 (ammonia), 6 (nitrogen oxides), 7 (alcohols), 8 (broad-spectrum volatile organic compounds), 9 (epoxides), 10 (aldehydes and alcohols, low threshold, high sensitivity), 11 (compounds responsive to photoionization detector), 12 (esters and ethers), 13 (aldehydes and ketones), 14 (chlorinated phenols), 15 (thiophenols), 16 (phenols), and 17 (amino-aromatic and nitrophenolic compounds). The sensor response was expressed as resistivity (Ohm).

For measurement, 0.5 g of PJH was placed into a 10 mL headspace vial. A total of 270 samples were analyzed (90 batches per storage year) using static headspace sampling. The operational parameters were set as follows: sensor flushing time, 80 s; sensor zeroing time, 5 s; sample preparation time, 5 s; injection flow rate, 400 mL·min^−1^; and data acquisition time, 70 s. Orthogonal partial least squares-discriminant analysis (OPLS-DA) was applied for pattern recognition of the flavor fingerprint data to establish a storage period discrimination model.

### 2.5. Analysis of Color and Apparent Quality of PJH with Different Storage Periods

A HySpex series hyperspectral imaging system (Norsk Elektro Optikk, Oslo, Norway) was used to collect spectral image data of PJH samples for the non-destructive evaluation of changes in their apparent quality. Two hundred batches were analyzed for each storage year, totaling 600 batches.

In the experiment, the imaging geometric parameters were strictly controlled: the object distance between the lens and the sample was fixed at 30 cm, a push-broom linear scanning mode was adopted, and the system spectral resolution was 6 nm. The experiment was equipped with a dual-band hyperspectral camera covering the visible-near-infrared (VNIR, 410–990 nm) and short-wave infrared (SWIR, 950–2500 nm) spectral regions, which collected 108 and 288 effective spectral channels, respectively. After field of view (FOV) calibration, the exposure integration time and frame rate cycle of the VNIR lens were set to 3500 μs and 15,000 μs, respectively, while those of the SWIR lens were optimized to 3000 μs and 39,107 μs to ensure the consistency of imaging signal-to-noise ratio (SNR) across different spectral regions.

After the collection of hyperspectral image data, the original hyperspectral images were subjected to RAD correction using the software (HySpex RAD; Norsk Elektro Optikk, Oslo, Norway) provided with the instrument. Subsequently, flat field correction was performed using ENVI 5.3 software. The correction formula is as follows:

(1) $$\begin{array}{c} R=\left(I_{R}-I_{B}\right)/\left(I_{W}-I_{B}\right)\; \end{array}$$

where *R* is the corrected reflectance image, *I_R_* is the original reflectance image, *I_W_* is the whiteboard image, and *I_B_* is the blackboard reference image. After correction, the average relative reflectance spectra of the Region of Interest (ROI) of each sample were extracted to construct the original spectral dataset for subsequent analysis.

### 2.6. Construction of an Intelligent Discrimination Model for Storage Period Based on Hyperspectral Information

To establish a rapid and non-destructive identification method for the storage period of PJH, three ML algorithms were employed in this study to construct discrimination models based on hyperspectral data. PLS-DA performs dimensionality reduction and classification by extracting latent variables most relevant to the storage period; SVM aims to find the optimal classification hyperplane in the feature space; RF improves model robustness by integrating the prediction results of multiple decision trees.

To optimize model performance, five spectral preprocessing methods were systematically compared: Multiplicative Scatter Correction (MSC), Savitzky–Golay (SG) smoothing, Standard Normal Variate (SNV) transformation, First Derivative (FD), and Second Derivative (SD). These methods were used to eliminate baseline drift, enhance feature resolution, and thereby improve the prediction accuracy and stability of the models [27]. In addition, introducing a data fusion strategy to conduct a more comprehensive analysis of hyperspectral data enhances the accuracy and reliability of the model [28].

### 2.7. Data Analysis and Model Validation

All statistical graphs and tables were generated using GraphPad Prism 8.0.2 software, and inter-group differences were tested by One-way Analysis of Variance (One-way ANOVA). Prior to performing one-way ANOVA, all quantitative data were tested for normality (Shapiro–Wilk test) and homogeneity of variances (Bartlett’s test and Brown-Forsythe test). After confirming that all prerequisites were met, one-way ANOVA was performed for inter-group difference analysis, ensuring the reliability and scientificity of the statistical results. Machine learning modeling and computation were performed on the MATLAB 2022b platform.

The entire sample set was randomly divided into a training set and a test set at a ratio of 7:3. Ten-fold cross-validation was used to evaluate model performance, with Overall Accuracy (OA), Average Accuracy (AA), Single-Class Accuracy (SA), and Kappa coefficient as evaluation indicators. The Kappa coefficient measures the consistency between the model’s classification results and the actual situation, with values closer to 1 indicating better consistency. A grid search strategy was used to optimize model hyperparameters, and the optimal model combination was finally determined. The calculation formula is as follows:

(2) $$\begin{array}{c} {SA}_{i}={TP}_{i}/\left({TP}_{i}+{FN}_{i}\right) \end{array}$$

(3) $$OA=\sum_{i=1}^{C}{TP}_{i}/\sum_{i=1}^{C}\left({TP}_{i}+{FP}_{i}+{FN}_{i}+{TN}_{i}\right)\;$$

(4) $$AA=(1/C)\times \sum_{i=1}^{C}{SA}_{i}$$

(5) $$\begin{array}{c} Kappa=\left(OA-PE\right)/\left(1-PE\right)\; \end{array}$$

(6) $$PE=\sum_{i=1}^{C}\left\{\left[\left({TP}_{i}+{FP}_{i}\right)/N\right]\times \left[\left({TP}_{i}+{FN}_{i}\right)/N\right]\right\}$$

where *N* is the total number of samples, *C* is the total number of categories, *TP_i_* is the number of True Positives for the *i*-th category, *FN_i_* is the number of False Negatives for the *i*-th category, *FP_i_* is the number of False Positives for the *i*-th category, and *TN_i_* is the number of True Negatives for the *i*-th category. Expected Agreement (*PE*) refers to the theoretical accuracy of random classification.

## 3. Results and Discussion

### 3.1. Functional Activity of PJH with Different Storage Periods

As a functional food raw material, the anti-inflammatory and antioxidant functional activity of PJH is one of its core values. In this study, RAW264.7 murine macrophages were used as an in vitro inflammation model to evaluate the cell compatibility and anti-inflammatory function of PJH extracts from different storage periods, providing data support for food raw material shelf-life management and functional retention.

#### 3.1.1. Evaluation of Biocompatibility

**Figure 1:**
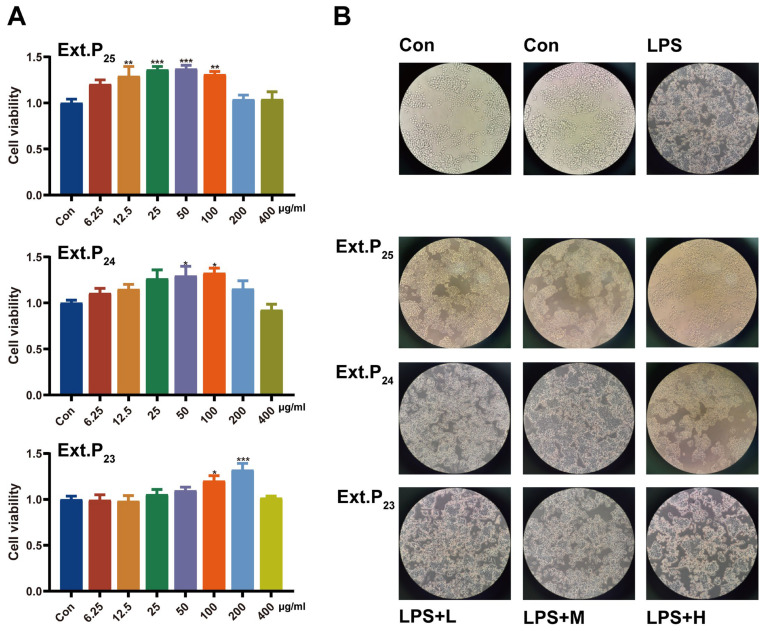
Cell viability and morphology under treatment with extracts from Pianjianghuang with different storage years. (**A**) Cell viability of the Ext.P_25_, Ext.P_24_, and Ext.P_23_ treatment groups, respectively (*n* = 6), note: mean ± SD. Intragroup comparison: * *p* < 0.05, ** *p* < 0.01, *** *p* < 0.001 vs. control group; (**B**) Typical morphology of RAW264.7 cells in different treatment groups.

As shown in Figure 1A, within the concentration range of 6.25–400 μg/mL, all three extracts exhibited no cytotoxicity (no significant decrease in cell viability compared with the blank control group). At low to medium concentrations (e.g., 6.25–100 μg/mL), some extracts showed a tendency to enhance cell viability, among which Ext.P_25_ significantly increased the cell survival rate at 12.5 μg/mL (*p* < 0.01). This indicates that samples from all years have good biocompatibility within the experimental concentration range and are suitable for subsequent functional activity studies.

Notably, the cell viability-promoting effect at 200–400 μg/mL decreased compared with those at lower concentrations (12.5–100 μg/mL). This phenomenon is possibly caused by the following reasons: First, multiple bioactive components (e.g., terpenes and curcuminoids) in the plant extract may enhance cell metabolism and proliferation by activating cellular protective pathways at low concentrations; however, when the concentration exceeds a certain threshold, these components reach saturation in the cellular microenvironment, and their proactive signals weaken, leading to a plateau or slight decline in the promoting effect. Second, the addition of high-concentration extracts may slightly alter the osmotic pressure of the culture medium. Although this osmotic pressure change does not reach the level of cell damage, it exerts certain effects on the proliferation rate of cells, resulting in a slight decrease in cell viability compared with the low to medium concentration groups. It should be emphasized that the cell viability at 200–400 μg/mL was not significantly lower than that of the control group, indicating satisfactory cell viability.

#### 3.1.2. Anti-Inflammatory Effect

Morphology regulation: LPS stimulation induced inflammatory morphological changes in RAW264.7 cells (increased volume, elongated pseudopodia), while all PJH extracts improved cell morphology in a concentration-dependent manner (Figure 1B). The protective effect followed Ext.P_25_ > Ext.P_24_ > Ext.P_23_, with Ext.P_25_ exerting efficacy at low concentrations and Ext.P_23_ only at high concentrations.

**Figure 2:**
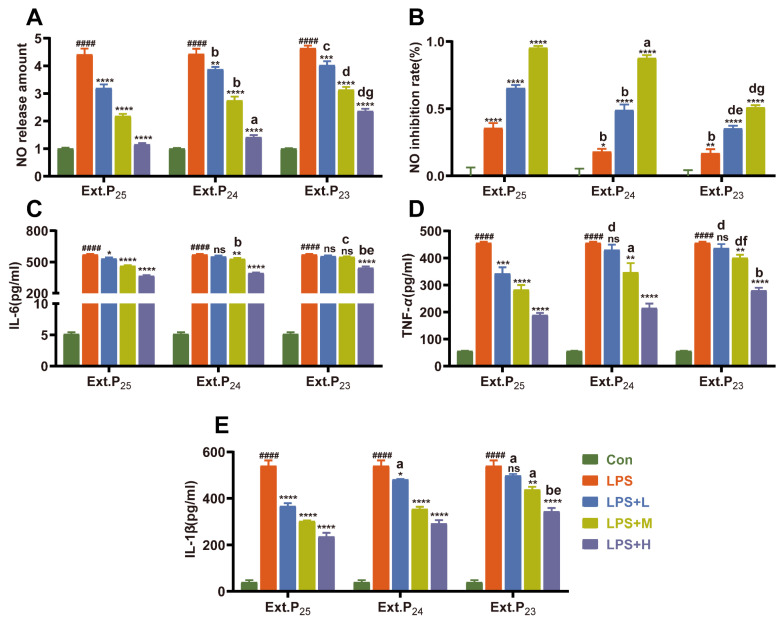
Anti-inflammatory and antioxidant activities of different extracts. (**A**) Effects of different extracts on NO release levels (*n* = 6); (**B**) NO inhibition rates of the three extracts (*n* = 6); (**C**) Effects of the three extracts on IL-6 release levels (*n* = 3); (**D**) Effects of the three extracts on TNF-*α* release levels (*n* = 3); (**E**) Effects of the three extracts on IL-1*β* release levels (*n* = 3). Note: mean ± SD. Intragroup comparison: *^####^ p* < 0.0001 vs. control group; * *p* < 0.05, ** *p* < 0.01, *** *p* < 0.001, **** *p* < 0.0001 vs. LPS model group, ns: *p* > 0.05 vs. LPS model group. Intergroup comparison (at the same dose): a–d vs. Ext.P_25_ (a, *p* < 0.05; b, *p* < 0.01; c, *p* < 0.001; d, *p* < 0.0001); e–g vs. Ext.P_24_ (e, *p* < 0.05; f, *p* < 0.01; g, *p* < 0.0001).

Inhibition of inflammatory mediators and cytokines: All extracts dose-dependently inhibited LPS-induced excessive NO release, with inhibitory effects negatively correlated with storage time (Figure 2A,B). At 50 μg/mL, Ext.P_25_ achieved a 95.39% NO inhibition rate (*p* < 0.0001), followed by Ext.P_24_ (87.73%, *p* < 0.0001) and Ext.P_23_ (51.01%, *p* < 0.0001). For pro-inflammatory cytokines (TNF-*α*, IL-6, IL-1*β*), Ext.P_25_ exhibited the strongest regulatory activity (Figure 2C–E): it significantly inhibited all three cytokines at 12.5 μg/mL (inhibition rates: 24.9%, 6.71%, 32.13%), with maximum inhibition rates of 52.66%, 33.25%, and 45.95% at 50 μg/mL. Ext.P_24_ only inhibited IL-1*β* at low concentrations and showed weaker overall activity, while Ext.P_23_ had no significant effects at low concentrations and only moderate inhibition at 50 μg/mL.

The collective results establish a clear negative correlation between PJH’s anti-inflammatory activity and storage time (Ext.P_25_ > Ext.P_24_ > Ext.P_23_).

### 3.2. Variation Rules of Functional Components in PJH with Different Storage Periods

**Figure 3:**
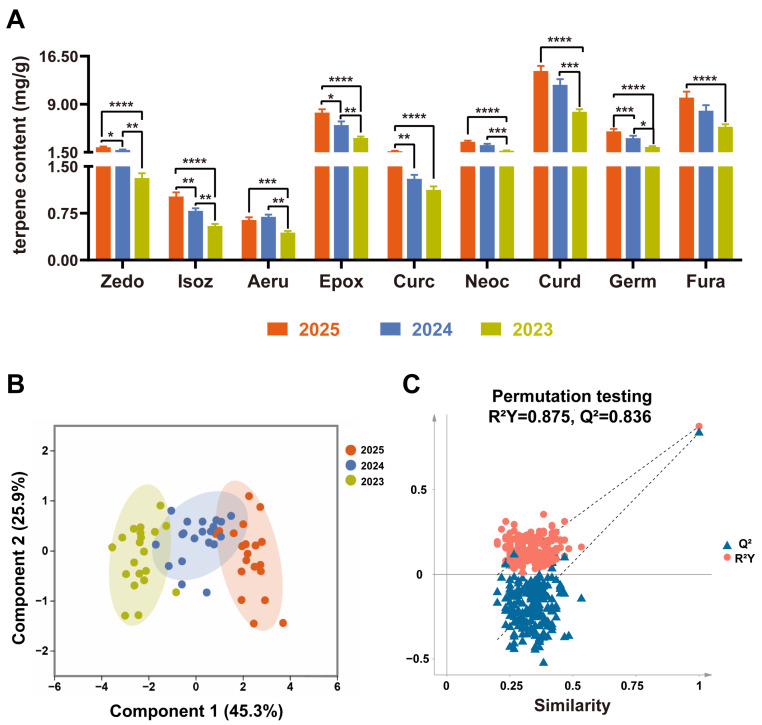
Terpene content in Pianjianghuang with different storage years. (**A**) Comparison of the contents of 9 terpenes (*n* = 20, mean ± SD, * *p* < 0.05, ** *p* < 0.01, *** *p* < 0.001, **** *p* < 0.0001); (**B**,**C**) OPLS-DA classification and Kruskal–Wallis test results based on the contents of 9 terpenes. Note: R^2^Y (proportion of variance explained in Y) = 0.875, Q^2^ (predictive ability) = 0.836; the *x*-axis “Similarity” denotes the permutation level of storage-year related data (1 corresponds to original unpermuted data); R^2^Y and Q^2^ increase significantly only for the original data, indicating that the model can effectively distinguish samples from different storage years. The same notations apply below.

The discrepancy in anti-inflammatory efficacy suggests a difference in terpene contents, which are the major contributors. Terpenes directly relate to the flavor quality and application value of PJH as a functional food raw material. The content dynamics of nine key terpenes across three storage years were analyzed using HPLC to elucidate the material basis underlying the observed functional deterioration (Figure 3A). The standard curve and content data are shown in Tables S1 and S2.

#### 3.2.1. Overall Variation Trend of Terpene Components

The results showed that the vast majority of terpene components exhibited a significant time-dependent decrease. To comprehensively evaluate the impact of storage periods on the chemical characteristics of PJH, Orthogonal Partial Least Squares-Discriminant Analysis (OPLS-DA) was employed for multivariate pattern recognition. As shown in Figure 3B,C, samples from different years formed distinct and independent clusters in the score plot, enabling accurate discrimination of storage years. The cumulative contribution rate of Component 1 and Component 2 reached 71.2%, indicating that the model is robust, reliable, and efficient in capturing the core variation mainly derived from storage time.

#### 3.2.2. Attenuation Characteristics of Key Components and Screening of Chemical Markers

The microscopic basis of this classification rule lies in the content changes in each terpene component. Comparing the 2025 and 2023 samples, the attenuation amplitudes of key components such as zedoarondiol, germacrone, and (4*S*,5*S*)-germacrone-4,5-epoxide all exceeded 42% (maximum 51.47%). This large-scale and high-intensity component attenuation is the direct material basis for the OPLS-DA model to achieve year-specific cluster separation; the significant negative correlation between component contents and storage time indicates that these terpenoid components can be used as objective chemical indicators for evaluating the storage quality of PJH.

Although the overall trend is decreasing, there are obvious differences in the degradation rates and stability of different components. Zedoarondiol, isozedoarondiol, germacrone, etc., showed significant decreases in pairwise comparisons of adjacent years, demonstrating high sensitivity to storage conditions and making them suitable as characteristic markers for changes in the freshness of food raw materials. Notably, the content of aerugidiol increased instead of decreasing from 2025 to 2024. This abnormal phenomenon suggests that the storage process of PJH is not merely simple component degradation but may involve complex chemical transformations (e.g., precursor components or isomers converted into aerugidiol through enzymatic/non-enzymatic reactions), and the generated amount temporarily offsets its own degradation loss.

This strategy of “single-component quantification (HPLC) + multivariate pattern recognition (OPLS-DA)” systematically clarified the impact of storage periods on the chemical composition of PJH from both single components and overall chemical profile, verifying the consensus that fresh means superior efficacy.

### 3.3. Analysis of Flavor Quality Evolution of PJH Based on E-Nose Technology

#### 3.3.1. Correlation Between E-Nose Sensor Responses and Volatile Flavor Compounds

**Figure 4:**
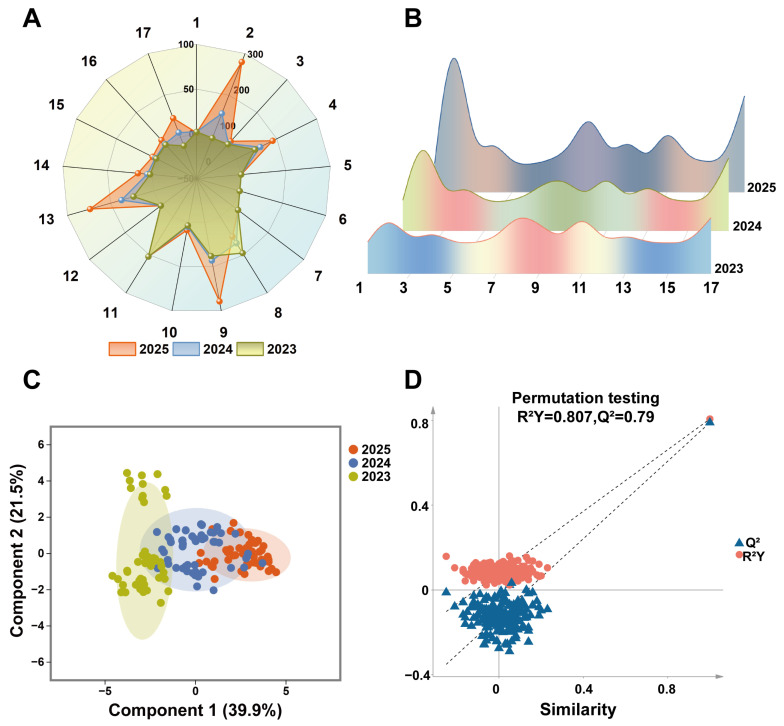
Sensory characterization of Pianjianghuang with different storage years using an electronic nose. (**A**,**B**) Visual characterization of odor (radar chart and ridge plot) of PJH with different storage years; 1 (olefins), 2 (aldehydes and alcohols, high threshold), 3 (organic sulfides), 4 (aldehydes and alcohols, medium threshold), 5 (ammonia), 6 (nitrogen oxides), 7 (alcohols), 8 (broad-spectrum volatile organic compounds), 9 (epoxides), 10 (aldehydes and alcohols, low threshold, high sensitivity), 11 (compounds responsive to photoionization detector), 12 (esters and ethers), 13 (aldehydes and ketones), 14 (chlorinated phenols), 15 (thiophenols), 16 (phenols), and 17 (amino-aromatic and nitrophenolic compounds); Note: In the E-nose odor radar chart (**A**), the axis range of sensors 1 and 3−17 is set at −50 to 100, while that of sensor 2 is 0 to 300; (**C**,**D**) OPLS-DA classification model and Kruskal–Wallis test results based on E-nose data.

This study adopted electronic nose technology for the digital characterization of flavor profiles of PJH. Radar chart analysis (Figure 4A,B) intuitively presented the flavor contour of PJH. The results showed that Sensors 2, 17, 11, 13, 8, 9, and 4 exhibited high response values to PJH samples with different storage years. Combined with the sensitivity characteristics of the sensors, it was confirmed that the main volatile flavor components of PJH were aldehydes, alcohols, and phenols.

Specifically, the synergistic high responses of Sensor 2 (high-threshold sensitivity to aldehydes/alcohols), Sensor 4 (medium-threshold sensitivity to aldehydes/alcohols), Sensor 7 (sensitivity to alcohols), and Sensor 10 (low-threshold and high-sensitivity to aldehydes/alcohols) verified that aldehydes and alcohols are the core flavor substances of PJH. Most of these components have fresh, fruity, or spicy aromas, which is consistent with the literature that volatile components of Zingiberaceae plants are mainly terpenes, aldehydes, and alcohols. It also aligns with the characteristics of Zingiberaceae plants as important spices.

The presence of broad-spectrum volatile organic compounds (Sensor 8, 11) and characteristic aromatic components (Sensor 16, 17) was also detected, reflecting the flavor complexity of PJH as a functional food raw material. Notably, with the extension of storage time, the response values of each sensor showed a systematic downward trend, indicating significant volatilization or chemical transformation of the characteristic flavor components of PJH during storage. This is consistent with the practice that aged materials have a weaker flavor.

#### 3.3.2. Establishment and Validation of Flavor Fingerprint Model

To accurately analyze the subtle flavor differences in samples with different storage years, OPLS-DA was used for dimensionality reduction in electronic nose data, and a discriminant model for the storage time of PJH was established (Figure 4C). The score plot showed good clustering separation of samples from different years: Principal Component 1 (explaining 39.9% of the variance) represented flavor changes related to storage time, and Principal Component 2 (explaining 21.5% of the variance) reflected individual differences among samples. The cumulative explanatory rate of the model reached 61.4%, and permutation test results (R^2^ = 0.807, Q^2^ = −0.79) confirmed that the model was robust and reliable without overfitting (Figure 4D). This model achieves accurate and rapid identification of PJH storage years, and compared with traditional sensory evaluation and chromatographic analysis, it has the advantages of objectivity, efficiency, and non-destructiveness, providing an effective technical means for quality monitoring of functional food raw materials in the food industry.

### 3.4. Hyperspectral Data Analysis of PJH with Different Storage Periods

#### 3.4.1. Overall Spectral Trend and Stability of Core Components in Food Raw Materials

**Figure 5:**
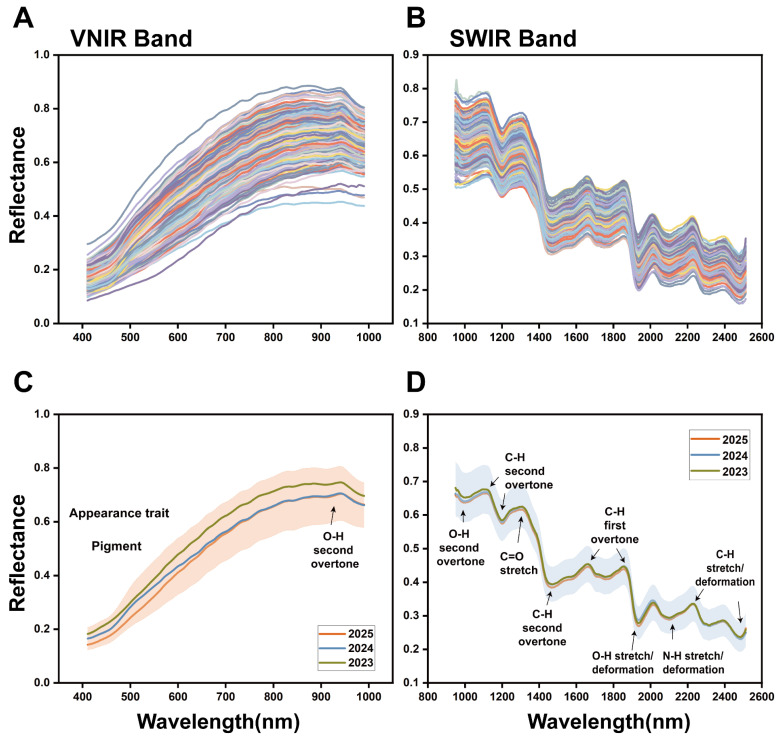
Color, internal/external traits, and hyperspectral features of Pianjianghuang with different storage years. (**A**) Hyperspectral original spectral curves of PJH in the VNIR band. (**B**) Hyperspectral original spectral curves of PJH in the SWIR band. (**C**) Average spectral curves and characteristic peak analysis in the VNIR band. (**D**) Average spectral curves and characteristic peak analysis in the SWIR band.

The overall spectral profiles of PJH samples across three storage years exhibited consistent peak positions and shapes (Figure 5A,B). This indicates that the fundamental molecular scaffolds, characterized by specific chemical bonds (e.g., C–H, O–H), remained intact during storage. However, this spectral consistency should not be misinterpreted as stability in the content of key functional components. Significant differences in the average reflectance were observed, which are attributed to alterations in the PJH’s physical structure (e.g., particle packing, porosity) and, more critically, to the substantial decrease in the concentration of light-absorbing functional compounds (e.g., terpenes), as quantitatively confirmed by our HPLC analysis (Section 3.2). These combined physical and chemical changes modulate the absorption-scattering properties of PJH, providing a direct physicochemical basis for the successful discrimination of storage years using HSI.

#### 3.4.2. Analysis of Color Quality in the VNIR Band

In the VNIR range, reflectance systematically increased with storage time (Figure 5C), visually corresponding to sample fading. This is mechanistically linked to the oxidative degradation of curcuminoids and other pigmented terpenes, which reduces visible light absorption and increases reflectance, directly quantifying changes in PJH’s appearance traits and pigment stability, providing an intuitive indicator for raw material freshness evaluation.

#### 3.4.3. Characterization of Intrinsic Components and Storage Quality Deterioration in SWIR Band

Within the 990–1350 nm range (Figure 5D), the spectrum forms a distinct high-reflectance region with characteristic absorption peaks at 990 nm, 1100 nm, 1200 nm, etc. At 990 nm, the O–H stretching vibration second overtone (polysaccharides, moisture) is observed. Increased peak intensity suggests moisture absorption during storage, which induces mold growth, accelerates functional component degradation, and promotes hydrolysis of cell wall polysaccharides (cellulose, pectin). These changes weaken cell wall mechanical strength, increase physical damage susceptibility, and shorten shelf life [27,29]. At 1100/1200 nm, the C–H stretching vibration second overtones are observed; at 1450 nm, the peak is characteristic of polysaccharides, non-volatile acids, and terpenoids. Reflectance differences here reflect changes in structural (cellulose) and bioactive (terpene) component contents. Cellulose conformation/aggregation changes alter light scattering, while accumulation of polysaccharide degradation products (e.g., reducing sugars) and non-volatile acids contributes to late-storage texture softening and flavor changes [30]. At 1300 nm, the C=O stretching vibration peak (terpenes, aromatic conjugated carbons) is observed. Peak changes indicate terpenoid oxidation (e.g., ketone/aldehyde group formation), confirming functional component degradation and explaining weakened bioactivity [31].

In the 1662–2500 nm band, the chemical assignment of characteristic peaks focuses more on the functional/nutritional components and storage-related safety changes in PJH. At 1662/1850 nm, the C–H stretching vibration first overtones are observed; at 2200/2500 nm, the C–H stretching/bending combination peaks (–CH=CH groups) are observed. These correlate with terpenes and flavonoids, with peak shifts indicating structural modification or mild degradation of curcuminoids and terpenes (e.g., curcumol) [32,33]. At 1935 nm, the O–H stretching/bending combination peak (polysaccharides, OH-containing compounds) is observed. This complements the 990 nm band results, reinforcing evidence of raw material deterioration [21,30]. At 2112 nm, the N–H/–NH_2_ characteristic absorption (proteins, amino acids) peak is observed. Peak changes suggest Maillard reactions or mild hydrolysis during storage, which affect flavor (e.g., producing off-odors) and may involve safety risks (e.g., advanced glycation end products accumulation), providing a new perspective for storage safety control [34,35].

In summary, the systematic reflectance variations in both VNIR and SWIR bands quantitatively capture the storage-induced deterioration of PJH. Specific wavelengths associated with pigments, terpenes, moisture, and polysaccharides (e.g., 990, 1300, 1450, 1935 nm) serve as optimal features for rapid, non-destructive storage period discrimination.

### 3.5. Rapid and Non-Destructive Shelf-Life Intelligent Discrimination of PJH Based on HSI-ML

To meet the high-throughput and non-destructive quality control requirements of the food industry for the storage quality of raw material for functional food ingredients, this study systematically compared three ML algorithms, SVM, RF, and PLS-DA, combined with spectral data fusion strategies and preprocessing methods, to analyze the adaptability of the models to the HSI data of PJH during storage.

#### 3.5.1. Performance Differences and Mechanism Analysis of ML Models

The three models exhibited significant performance differences (Table 1), primarily attributable to their inherent capacities to extract subtle, storage-related spectral features from high-dimensional data.

The RF model demonstrated limited efficacy (OA/AA ≤ 71.67%; Kappa ≤ 0.575), struggling with the high dimensionality and low signal-to-noise ratio of the HSI data. Its ensemble decision-tree structure is susceptible to irrelevant spectral variations (e.g., baseline drift), which overshadowed the gradual chemical changes indicative of storage time. Similarly, the SVM model with a radial basis function (RBF) kernel failed to achieve high accuracy post-hyperparameter optimization. The RBF kernel is well-suited for non-linear data with distinct feature boundaries, but PJH’s spectral differences arise from continuous, gradual changes in functional component contents—resulting in fuzzy, gradient feature boundaries. This mismatch limits the kernel’s ability to accurately characterize classification boundaries, failing to address the “continuous gradient discrimination” demand for food shelf-life assessment.

In contrast, the PLS-DA model achieved the best performance, with OA/AA > 97% and Kappa > 0.97 in both bands. Its superiority stems from its supervised dimensionality reduction mechanism: PLS-DA explicitly constructs a linear projection between spectral variables and the target “storage period”, aligning with the “multivariate correlation” nature of hyperspectral data. During dimensionality reduction, it prioritizes extracting spectral features strongly correlated with storage period (e.g., SWIR band peaks associated with sesquiterpenoid hydroxyl/methyl vibrations) while effectively suppressing irrelevant noise (e.g., scattering from raw material surface residues, instrument errors). This exactly matches PJH’s storage characteristics (subtle chemical changes—weak spectral responses), providing reliable support for precise shelf-life discrimination.

#### 3.5.2. Optimization of Spectral Data Processing and Final Model Selection

Most models performed better in the SWIR band than the VNIR band, reflecting the internal-external aspect of food raw material quality determination: the SWIR band carries combination/overtone absorption information of O–H, C–H, and N–H functional groups, directly reflecting molecular vibration and structural changes in intrinsic components (terpenes, polysaccharides). Storage-induced component oxidation, degradation, and migration are accurately captured as characteristic spectral responses, making it an ideal “intrinsic quality fingerprint carrier”; the VNIR band focuses on surface physical properties (color, texture) that are easily interfered with by light and surface contamination. PJH’s gradual color change during storage is visually indistinguishable, resulting in low discriminative information content and limited model performance. Data-level fusion of VNIR and SWIR bands did not yield a synergistic improvement in model performance (Table 2). This indicates significant information overlap between the two bands and suggests that the SWIR spectrum alone provides sufficient discriminative features for a robust model like PLS-DA.

**Figure 6:**
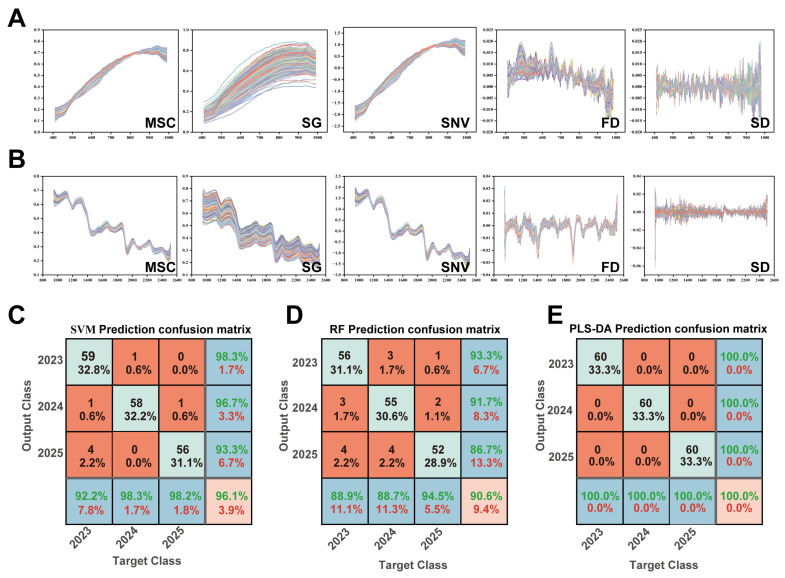
Optimized results from different spectral preprocessing methods and prediction performance using machine learning models. (**A**) Spectral preprocessing result curves in the VNIR band; (**B**) Spectral preprocessing result curves in the SWIR band; (**C**) Optimal result of the SVM model (based on full-band data preprocessed by FD); (**D**) Optimal result of the RF model (based on full-band data preprocessed by FD); (**E**) Optimal result of the PLS-DA model (based on SWIR band data preprocessed by FD/SD).

Data preprocessing is a crucial link in improving the performance of hyperspectral models (Figure 6A,B). The results of this study show that the impact of preprocessing methods on model performance exhibits significant spectral band and model type dependence (Table 1 and Table 2). VNIR band: MSC/SNV transformations only improved SVM/RF accuracy by only 3.89%~7.77%. FD preprocessing increased the RF model’s accuracy to 82.22% (Kappa 73.33%), but it showed no obvious gain for the PLS-DA. Limited improvement stems from weak correlation between VNIR surface information and storage period—preprocessing can only eliminate partial instrument noise and surface interference, not extract additional effective features. FD effectively alleviates RF’s noise sensitivity by amplifying weak signals. SWIR band: Preprocessing (MSC/SNV/FD/SD) significantly optimized model performance (except SG smoothing for RF). Notably, FD/SD preprocessing enabled PLS-DA to achieve 100% in OA/AA, and the Kappa coefficient (Figure 6E), realizing perfect storage period discrimination. This is because SWIR’s rich molecular fingerprint information is enhanced by FD/SD—baseline drift is eliminated, and fine functional group vibration features (e.g., curcuminoid/terpene characteristic peaks) are highlighted, providing high-discriminative input for PLS-DA. In the full-band range, FD preprocessing also enhanced the performance of SVM/RF. This confirms that there is still effective information in the full-band data that has not been fully utilized by a single band, and targeted preprocessing can synergistically release the potential value of multispectral data.

Comprehensive consideration of model performance, spectral information characteristics, and preprocessing optimization effects led to the final selection of the strategy “SWIR band + FD/SD preprocessing + PLS-DA model” to construct the shelf-life discrimination model for PJH.

### 3.6. Discussion

This study systematically demonstrates that prolonged storage leads to a significant decline in the bioactivity of PJH, a finding that underscores the importance of shorter storage periods for preserving its functional value. The observed reduction in anti-inflammatory efficacy is fundamentally attributed to the degradation of key functional components. Quantitative analysis by HPLC revealed that the contents of 9 key terpene components in PJH showed a significant decreasing trend over storage periods, likely attributable to the degradation of light and oxygen environmental factors sensitive bioactive compounds, such as terpenes and curcuminoids. Notably, although samples were stored in a light-proof environment to minimize light exposure (consistent with conventional industrial practices), trace ambient diffused light remains unavoidable in practical circulation. Terpenes, as well as curcuminoids, are inherently sensitive to such environmental stimuli: terpenes in particular possess conjugated double bond structures that render them photosensitive, making photo-degradation a non-negligible factor affecting their stability during long-term storage. The differential inhibition patterns of cytokines further suggest that specific bioactive constituents degrade at varying rates, highlighting the complexity of storage-induced compositional changes. These results are consistent with previous reports on the storage-induced quality deterioration in functional food materials [36].

This functional and chemical degradation was further reflected in PJH’s sensory attributes: E-nose detected the attenuation of characteristic aromas, while HSI captured color fading. E-nose-derived flavor fingerprints established an accurate correlation between macroscopic storage-related quality changes and microscopic volatile profiles, enabling the non-destructive assessment of raw material component integrity and functional potential. Meanwhile, HSI captured subtle variations in macroscopic appearance (color) and linked these to the dynamic evolution of intrinsic functional/nutritional components via characteristic spectral peak changes—with the VNIR band reflecting surface traits (e.g., color) and the SWIR band characterizing intrinsic chemical composition, enabling a “surface-to-interior” characterization of PJH. Collectively, storage-induced synergistic changes in volatile flavor components and non-volatile functional ingredients drive the systematic decline of PJH’s comprehensive quality, which echoes the terpene degradation data and constructs a holistic “sensory characteristics–chemical components–functional activity” evaluation system. This multidimensional quality deterioration lays the material foundation for developing a rapid quality assessment method.

Based on the material basis underlying the multidimensional quality changes in PJH during storage, an HSI-ML model was proposed for the intelligent discrimination of PJH shelf life, and the performance of three classic machine learning models (RF, SVM, and PLS-DA) was compared using hyperspectral data. Specifically, the RF model, being sensitive to high-dimensional and low signal-to-noise ratio data, struggled to capture subtle storage-related chemical changes; the SVM model had limitations in handling the continuous spectral changes caused by the gradual degradation of terpene components; while PLS-DA, by virtue of its supervised dimensionality reduction mechanism, could effectively establish a mapping relationship between spectral variables and storage periods, which is more consistent with the multivariate correlation characteristics of hyperspectral data and the gradual degradation features of PJH during storage.

Model optimization further revealed that the SWIR band alone provided more discriminative power than full-band fusion or the VNIR band, underscoring the practical advantage of “precision band selection” over “full-band fusion”. The results of data preprocessing experiments highlighted the necessity of establishing an adaptive framework of “spectral characteristics–model requirements–preprocessing methods”: the selection of preprocessing methods needs to comprehensively consider the physical significance of spectral regions (e.g., VNIR is dominated by scattering, while SWIR is characterized by chemical fingerprints) and the model’s sensitivity to noise/features (e.g., RF requires FD for denoising, and PLS-DA can leverage FD/SD to enhance the response of effective features).

Ultimately, this study proposes “SWIR spectroscopy + FD/SD preprocessing + PLS-DA modeling” as the optimal technical combination for PJH shelf-life monitoring. This selection is both scientific and practical for the food industry: it targets the fundamental chemical changes in PJH, employs a simple preprocessing step to enhance features, and achieves 100% accuracy, all while simplifying data acquisition and reducing computational cost for potential industrial application, making it easier to meet the industrial needs of food enterprises for high-throughput screening of raw material inbound and dynamic monitoring of shelf life.

## 4. Conclusions

In summary, this study demonstrates that storage duration is a critical determinant of the multidimensional quality deterioration of PJH, systematically validated through a holistic “bioactivity-chemical composition-sensory attributes” evaluation strategy, establishing a “volatile-component-centered” causal chain linking intrinsic quality and apparent traits. Prolonged storage led to a significant decline in bioactivity and a corresponding loss of key terpene components. This functional and chemical degradation was intrinsically linked to a decline in sensory quality, with E-nose and HSI capturing the attenuation of characteristic aromas and the fading of color, respectively. This paradigm clarifies the intrinsic correlation between these indicators by converting subjective sensory characteristics into objective spectral/E-nose data, illustrating a non-destructive, high-throughput analytical approach to characterize the dynamic changes in volatile and non-volatile functional components during storage.

Notably, this study innovatively demonstrated that macroscopic sensory quality can serve as a quantitative indicator for intelligent grading. The PLS-DA model constructed with SWIR hyperspectral data after FD/SD preprocessing attained 100% accuracy in non-destructive discrimination of PJH storage years; this technical framework holds strong scalability for terpene-rich Zingiberaceae plants (e.g., *Curcuma longa* L. and *Zingiber officinale* Roscoe), given that their shared terpene profiles and storage-induced quality degradation mechanisms yield comparable spectral-sensory-chemical correlations to PJH. This establishes a rapid, non-destructive, and high-precision intelligent monitoring method, realizing a paradigm shift from “empirical judgment” to “data-driven quality control” for food raw materials—aligning with the modern food industry’s demand for standardized management. Moreover, this work holds critical guiding significance for promoting the standardization, intelligence, and precision of quality management in the functional food industry, especially for raw materials whose volatile components are the core of their nutritional and sensory value.

## Figures and Tables

**Table 1:** Performance of the three models based on VNIR and SWIR band HSI data on the test set.

Models	Band Range	Preprocessing Methods	SA(%)			OA (%)	AA (%)	Kappa × 100
			2023	2024	2025			
SVM	VNIR	RAW	90.00	85.00	90.00	88.33	88.33	82.50
		MSC	88.33	91.67	98.33	92.78	92.78	89.09
		SNV	85.00	96.67	95.00	92.22	92.22	88.33
		SG	90.00	86.67	88.33	88.33	88.33	82.50
		FD	83.33	63.33	90.00	78.89	78.89	68.33
		SD	55.00	48.33	40.00	47.78	47.78	21.66
	SWIR	RAW	83.33	86.67	66.67	78.89	78.89	68.33
		**MSC**	**95.00**	**100.00**	**88.33**	**94.44**	**94.44**	**91.67**
		SNV	91.67	91.67	83.33	88.89	88.89	83.38
		SG	86.67	88.33	70.00	81.67	81.67	72.50
		FD	90.00	100.00	81.67	90.56	90.56	85.83
		SD	86.67	98.33	55.00	80.00	80.00	69.14
RF	VNIR	RAW	70.00	76.67	68.33	71.67	71.67	57.50
		MSC	68.33	78.33	91.67	79.44	79.44	69.17
		SNV	70.00	73.33	95.00	79.44	79.44	69.17
		SG	70.00	76.67	76.67	74.44	74.44	61.67
		**FD**	**80.00**	**76.67**	**90.00**	**82.22**	**82.22**	**73.33**
		SD	88.33	48.33	66.67	67.78	67.78	51.66
	SWIR	RAW	51.67	66.67	40.00	52.78	52.78	29.16
		MSC	65.00	85.00	58.33	69.44	69.44	54.16
		SNV	60.00	81.67	65.00	68.89	68.89	53.33
		SG	45.00	63.33	36.67	48.33	48.33	22.50
		**FD**	**91.67**	**78.33**	**70.00**	**80.00**	**80.00**	**70.00**
		SD	70.00	73.33	66.67	70.00	70.00	55.00
PLS-DA	VNIR	RAW	93.33	98.33	100.00	97.22	97.22	97.13
		MSC	95.00	96.67	100.00	97.22	97.22	97.13
		SNV	93.33	98.33	100.00	97.22	97.22	97.13
		SG	96.67	98.33	98.33	97.78	97.78	97.70
		FD	96.67	98.33	100.00	98.33	98.33	98.28
		SD	**100.00**	**100.00**	**98.33**	**99.44**	**99.44**	**99.43**
	SWIR	RAW	95.00	98.33	100.00	97.78	97.78	97.70
		MSC	98.33	100.00	100.00	99.44	99.44	99.43
		SNV	93.33	98.33	100.00	97.22	97.22	97.13
		SG	98.33	100.00	100.00	99.44	99.44	99.43
		FD	**100.00**	**100.00**	**100.00**	**100.00**	**100.00**	**100.00**
		SD	**100.00**	**100.00**	**100.00**	**100.00**	**100.00**	**100.00**

The evaluation indicators include OA, AA, and the Kappa coefficient (Kappa × 100). The outstanding predictive performance is displayed in bold. The following is also applicable.

**Table 2:** Performance of the three models based on fused spectral data on the test set.

Models	Preprocessing Methods	SA(%)			OA(%)	AA(%)	Kappa × 100
		2023	2024	2025			
SVM	RAW	83.33	86.67	76.67	82.22	82.22	73.33
	MSC	96.67	90.00	88.33	91.67	91.67	87.50
	SNV	63.33	78.33	73.33	71.67	71.67	57.50
	SG	83.33	86.67	78.33	82.78	82.78	74.17
	FD	**98.33**	**96.67**	**93.33**	**96.11**	**96.11**	**94.17**
	SD	91.67	93.33	40.00	75.00	75.00	62.50
RF	RAW	70.00	75.00	53.33	66.11	66.11	49.17
	MSC	70.00	80.00	63.33	71.11	71.11	56.67
	SNV	68.33	76.67	60.00	68.33	68.33	52.50
	SG	65.00	75.00	53.33	64.44	64.44	46.67
	**FD**	**93.33**	**91.67**	**86.67**	**90.56**	**90.56**	**85.83**
	SD	76.67	68.33	73.33	72.78	72.78	59.17
PLS-DA	RAW	96.67	98.33	98.33	97.78	97.78	97.70
	MSC	96.67	98.33	100.00	98.33	98.33	98.28
	SNV	95.00	98.33	100.00	97.78	97.78	97.70
	SG	93.33	98.33	98.33	96.67	96.67	96.55
	FD	**100.00**	**100.00**	**98.33**	**99.44**	**99.44**	**99.43**
	SD	100.00	100.00	93.33	97.78	97.78	97.70

The evaluation indicators include OA, AA, and the Kappa coefficient (Kappa × 100). The outstanding predictive performance is displayed in bold.

## Data Availability

The original contributions presented in the study are included in the article/Supplementary Material, further inquiries can be directed to the corresponding authors.

## Supplementary Materials

The following supporting information can be downloaded at https://www.mdpi.com/article/10.3390/foods15020274/s1. Table S1: The contents of the key nine terpenes in different storage years; Table S2: Concentration of standard solution and corresponding peak area.
