# Supplementary material for: Investigation and Rapid Determination of Storage-Induced Multidimensional Quality Deterioration of Wenyujin Rhizoma Concisum
Source: Foods. 2026 Jan 12;15(2):274. doi: 10.3390/foods15020274 (PMC12839892; doi:10.3390/foods15020274)

Table S1

The contents of the key nine terpenes in different storage years

storage years	Content (mg/g)								
	1	2	3	4	5	6	7	8	9
2025	3.42	1.54	0.99	11.31	2.58	4.73	21.84	8.49	19.14
	2.08	0.90	0.50	6.58	1.50	3.02	11.63	4.31	10.40
	1.81	0.82	0.50	10.82	1.47	3.32	16.68	6.11	14.53
	2.78	1.26	0.82	7.25	2.01	3.14	15.25	4.47	10.27
	2.24	1.01	0.69	5.15	1.62	2.82	11.22	3.94	8.82
	2.06	0.89	0.45	10.80	1.46	3.40	16.91	5.17	5.40
	2.26	1.00	0.63	7.12	1.48	3.12	12.62	3.65	8.16
	1.35	0.59	0.35	5.99	0.99	1.97	9.59	3.62	7.97
	1.91	0.82	0.43	8.41	1.40	2.72	12.95	3.14	3.87
	3.44	1.58	1.06	8.34	2.48	4.22	18.28	7.21	15.73
	2.16	0.99	0.70	4.67	1.48	2.26	10.59	3.45	7.07
	2.53	1.15	0.73	11.07	1.89	4.41	18.78	6.81	16.20
	2.14	0.99	0.68	4.79	1.57	2.45	11.34	3.41	7.15
	1.93	0.85	0.62	6.01	1.23	2.60	10.58	2.92	5.44
	1.50	0.64	0.35	6.64	1.15	2.92	10.27	3.88	9.58
	2.15	0.96	0.60	5.79	1.52	2.65	13.10	4.11	9.94
	2.09	0.95	0.60	7.10	1.54	2.87	14.36	5.10	12.41
	2.19	0.96	0.60	6.15	1.52	2.80	11.52	3.64	9.02
	1.98	0.86	0.56	12.00	1.65	3.67	18.50	7.56	14.85
	3.50	1.64	0.97	7.88	2.44	3.75	17.69	4.33	4.60
Average	2.28	1.02	0.64	7.69	1.65	3.14	14.19	4.77	10.03
2024	1.68	0.70	0.66	4.59	1.17	2.39	10.49	3.02	7.36
	1.91	0.81	0.72	7.54	1.36	3.15	13.92	4.13	9.11
	1.90	0.83	0.80	4.30	1.40	2.29	11.06	3.13	7.11
	1.56	0.67	0.54	3.34	0.93	1.80	7.89	1.69	2.13
	1.95	0.83	0.76	7.91	1.45	2.91	15.67	4.97	9.95
	1.99	0.87	0.77	7.25	1.39	2.95	13.10	3.57	7.28
	1.72	0.75	0.68	4.50	1.24	2.17	9.92	2.89	6.21
	2.59	1.12	0.94	6.18	1.68	2.99	13.45	4.20	8.90
	1.64	0.68	0.68	6.50	1.18	2.63	13.01	3.86	9.01
	2.10	0.88	0.77	7.49	1.52	3.30	15.45	5.44	11.42
	1.46	0.60	0.47	2.99	1.00	1.69	7.34	1.95	4.46
	2.23	0.99	0.79	3.54	1.43	2.31	9.90	2.67	5.73
	1.91	0.83	0.63	3.91	1.24	2.25	9.43	3.07	7.51
	1.50	0.61	0.62	5.88	1.20	2.27	11.89	3.07	6.17
	1.85	0.77	0.57	2.46	1.17	1.74	8.33	2.49	5.67
	2.18	0.94	0.84	8.62	1.59	3.80	17.19	6.12	14.14
	2.44	1.06	0.91	13.47	1.90	5.59	23.50	8.95	19.57

	1.13	0.47	0.44	5.59	0.91	2.37	10.26	3.57	8.62
	2.16	0.92	0.88	5.40	1.52	2.36	12.24	3.22	5.49
	1.07	0.45	0.38	3.36	0.77	1.48	6.40	1.93	4.20
Average	1.85	0.79	0.69	5.74	1.30	2.62	12.02	3.70	8.00
2023	1.14	0.46	0.44	5.20	1.14	2.09	10.20	3.17	7.96
	1.55	0.63	0.55	3.07	1.26	1.73	7.65	2.25	5.24
	1.41	0.60	0.50	4.63	1.28	2.14	9.21	3.01	6.90
	1.04	0.43	0.34	3.51	0.97	1.56	7.10	2.34	6.39
	1.12	0.45	0.38	4.93	0.98	1.88	8.22	2.36	5.32
	0.78	0.31	0.22	2.64	0.69	1.06	4.53	1.28	3.18
	0.90	0.36	0.31	3.48	0.80	1.44	5.81	1.87	4.72
	1.03	0.38	0.37	5.62	0.97	1.76	9.12	2.74	6.20
	1.35	0.58	0.46	2.73	1.20	1.60	6.60	2.05	4.96
	1.50	0.61	0.45	3.26	1.18	1.86	8.22	1.82	2.93
	2.06	0.87	0.66	2.89	1.57	1.99	9.41	2.74	7.02
	1.32	0.56	0.44	3.07	1.12	1.58	6.62	2.04	4.74
	1.60	0.70	0.54	5.10	1.48	2.18	10.19	3.25	8.54
	1.12	0.45	0.37	3.16	0.91	1.47	6.53	1.79	4.56
	1.29	0.54	0.44	3.07	1.14	1.70	5.58	2.77	6.70
	1.23	0.49	0.38	3.46	0.89	1.53	6.91	1.67	3.27
	1.90	0.82	0.63	2.57	1.45	1.81	8.25	1.94	3.70
	1.43	0.59	0.45	2.75	1.23	1.42	7.56	2.38	5.49
	1.66	0.73	0.58	4.91	1.46	1.99	10.83	3.12	8.43
	0.87	0.35	0.30	4.63	0.78	1.71	8.00	1.88	3.20
Average	1.32	0.55	0.44	3.73	1.13	1.73	7.83	2.32	5.47

Noet: compound 1. Zedoarondiol, 2. Isozedoarondiol, 3. Aerugidiol, 4. (4S,5S)-germacrone-4,5epoxide, 5. Curcumenone, 6. Neocurdione, 7. Curdione, 8. Germacrone, 9. Furanodinene.

Table S2

Concentration of standard solution and corresponding peak area

content	Area
0.1	188784
0.2	376493
0.4	757638
0.8	1520762
1.6	3023955

The calibration curve established in this study for curdione was $y = 2.00 \times 10^6 x + 927.25$, with $R^2 > 0.9999$, indicating a highly reliable fitting effect. The linear range was 0.1–1.6 mg/mL.

Figure S1. Standard solubility curve of curdione

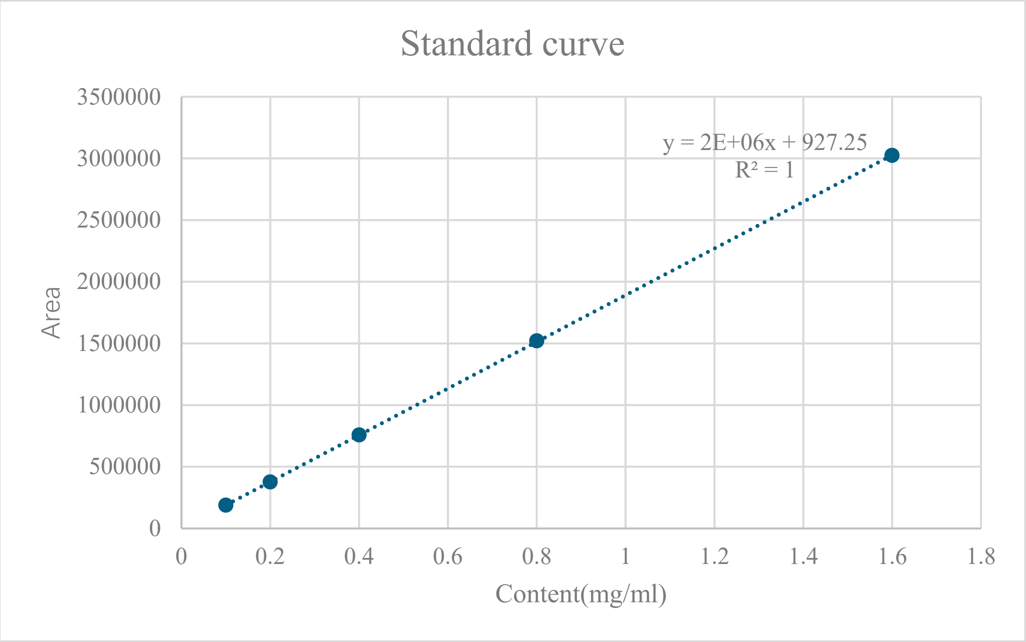

Supplement: Supplementary file 1 [file foods-15-00274-s001.zip › foods-4068458-supplementary.pdf]
